# Silencing of the lncRNA H19 enhances sensitivity to X-ray and carbon-ions through the miR-130a-3p /WNK3 signaling axis in NSCLC cells

**DOI:** 10.1186/s12935-021-02268-1

**Published:** 2021-12-04

**Authors:** Xueshan Zhao, Xiaodong Jin, Qiuning Zhang, Ruifeng Liu, Hongtao Luo, Zhen Yang, Yichao Geng, Shuangwu Feng, Chengcheng Li, Lina Wang, Xiaohu Wang, Qiang Li

**Affiliations:** 1 The First School of Clinical Medicine, Lanzhou University, Lanzhou, 730000 China; 2 Institute of Modern Physics, Chinese Academy of Sciences, Lanzhou, 730000 China; 3 University of Chinese Academy of Sciences, Beijing, 110000 China; 4 Lanzhou Heavy Ion Hospital, Lanzhou, 730000 China; 5 Affiliated Hospital of Qinghai University, Xining, 810000 China

**Keywords:** LncRNA H19, MiR-130a-3p, WNK3, Non-small-cell lung cancer, Radiotherapy

## Abstract

**Background:**

The lncRNA H19 is believed to act as an oncogene in various types of tumors and is considered to be a therapeutic target and diagnostic marker. However, the role of the lncRNA H19 in regulating the radiosensitivity of non-small cell lung cancer (NSCLC) cells is unknown.

**Methods:**

The expression profiles of lncRNAs in NSCLC were explored via transcriptome sequencing. CCK-8, EdU incorporation and clonogenic survival assays were conducted to evaluate the proliferation and radiosensitivity of NSCLC cells. Flow cytometry and Western blotting were conducted to measure the level of apoptosis. The binding relationship between the lncRNA H19 and miR-130a-3p was determined by a dual-luciferase reporter assay. A binding relationship was also identified between miR-130a-3p and With-No-Lysine Kinase 3 (WNK3).

**Results:**

Expression patterns of lncRNAs revealed that the lncRNA H19 was upregulated in radioresistant NSCLC (A549-R11) cells compared with A549 cells. Knockdown of the lncRNA H19 enhanced the sensitivity of NSCLC cell lines to X-ray and carbon ion irradiation. Mechanistically, the lncRNA H19 serves as a sponge of miR-130a-3p, which downregulates WNK3 expression. The lncRNA H19–miR-130a-3p–WNK3 axis modulates radiosensitivity by regulating apoptosis in NSCLC cell lines.

**Conclusion:**

Knockdown of the lncRNA H19 promotes the sensitivity of NSCLC cells to X-ray and carbon ion irradiation. Hence, the lncRNA H19 might function as a potential therapeutic target that enhances the antitumor effects of radiotherapy in NSCLC.

**Supplementary Information:**

The online version contains supplementary material available at 10.1186/s12935-021-02268-1.

## Introduction

The incidence of lung cancer ranks second among malignancies, just behind breast cancer. However, lung cancer is the most common cause of cancer death, causing 1.8 million deaths in 2020 [1]. The treatment modalities for lung cancer include surgery, radiotherapy, chemotherapy, targeted therapy, immunotherapy and so on. Despite the application of targeted therapy, immunotherapy and other treatment methods, the prognosis of lung cancer is very poor, with a 5-year survival rate varying from 4 to 17% [2]. Radiotherapy can be applied in all stages of disease. Our previous study showed that inspiring results were obtained with radiotherapy combined with immunotherapy [3]. However, radioresistance is acknowledged as a factor related to failure of cancer. It is crucial for us to identify key factors that lead to radioresistance and to improve the antitumor effects of radiotherapy. Combining radiation therapy with other approaches is a strategy for overcoming radioresistance and improving the tumor response to radiation in NSCLC. Hence, suitable therapeutic targets should be identified to help improve radiosensitivity.

Compared with photon radiotherapy, carbon ion radiotherapy (CIRT) has unique advantages, such as its Bragg peak, higher relative biological effect, higher linear energy transfer (LET), sharper dose distribution and better target conformity [4]. In particular, it was demonstrated to be safe for lung cancer patients with interstitial lung disease and older lung cancer patients [5, 6]. One recent prospective phase II study showed that the 2-year local control (LC) rate and overall survival (OS) rate were 91.2% and 91.9%, and the 5-year LC rate and 5-year OS rate were 88.1% and 74.9%, respectively, in patients with early-stage peripheral NSCLC [7]. The latest clinical outcome showed that the CIRT group had a higher 5-year LC rate (92.3% vs. 42.4%) and 5-year OS rate (71.8% vs. 34.4%) than the stereotactic body radiation therapy (SBRT) group in early-stage NSCLC [8]. Although CIRT can achieve certain results, there is still much room for improvement.

According to recent studies, dysregulation of the expression of microRNAs (miRNAs) and long noncoding RNAs (lncRNAs) can alter cellular radiosensitivity [9, 10]. LncRNAs, which contain > 200 nucleotides, and miRNAs, which contain approximately 22 nucleotides, are noncoding RNAs [11]. LncRNAs and miRNAs play significant roles in modulating the biological behaviors of tumors, such as proliferation and metastasis. For instance, the lncRNA CASC9.5 is involved in the proliferation and metastasis of lung adenocarcinoma (LUAD) [12]. The H19 gene, a 2.3 kb RNA molecule, is located on chromosome 11p15.5 [13]. The lncRNA H19 is believed to act as an oncogene in NSCLC and is considered to be a therapeutic target and diagnostic marker [14]. For example, a study revealed that the lncRNA H19 level is high in NSCLC patients and that this lncRNA may be a diagnostic marker; its diagnostic sensitivity and specificity were 67.74% and 63.08%, respectively [15]. In addition, single-nucleotide polymorphisms (SNPs) in the lncRNA H19 were found to be associated with susceptibility to lung cancer, especially NSCLC [16]. Numerous previous studies have confirmed that the lncRNA H19 is highly expressed in samples of lung cancer [17, 18]. To date, it remains unknown whether lncRNA H19 regulates radiosensitivity in NSCLC. This work focuses on the mechanism by which the lncRNA H19 regulates radiosensitivity in NSCLC.

## Materials and methods

### Cell culture

The human NSCLC lines A549 and H460 were grown in RPMI 1640 medium supplemented with 10% fetal bovine serum (FBS). Human embryonic kidney cells (HEK-293 T) were cultured in DMEM containing 10% FBS. These cell lines were purchased from the Cell Resource Centre of the Chinese Academy of Sciences and cultured at 37 °C in 5% CO_2_.

### Irradiation

X-rays were generated by an X-RAD generator (Faxitron, USA). The dose rate was 2.0 Gy/min (225 kV, 0.2 mm Al filter). Irradiation was also performed using a carbon ion beam (80.55 MeV/u) generated by the Deep Therapy Terminal, Institute of Modern Physics, Chinese Academy of Sciences. The irradiation parameters were as follows: dose rate of 2 Gy/min and broadened Bragg peak of 5 mm.

### RNA extraction and PCR

RNA extraction using TRIzol reagent (Sangon, China) was performed 24 h after transfection for RNA sequencing. Reverse transcription of mature miR-130a-3p was conducted with specific miRNA reverse transcription primers (RiboBio, China), and the internal reference was U6. For qRT–PCR analysis of mRNAs and the lncRNA H19, cDNA was synthesized with Prime Script RT Mix (Takara, China). The level of each mRNA and the lncRNA H19 was normalized to that of the GAPDH control. Changes relative to endogenous controls were calculated using the 2^−ΔCT^ method.

The primers used to amplify H19 were as follows:

Fw: 5′TCCTGAACACCTTAGGCTGG3′

Rev: 5′TGATGTTGGGCTGATGAGGT3′

The primers used to amplify WNK3 were as follows:

Fw: 5′TGTTGAAATGACGGAAGATGACA3′

Rev: 5′TCTGCCACTAGGAGAAGTAGC3′

### siRNA knockdown and miRNA mimic experiments

NSCLC cells were plated at 50% confluence in 35-mm Petri dishes. RiboFECTTM (RiboBio, China) was used as a transfection reagent. The concentration of both the miR-130a-3p mimic and si-H19 (RiboBio, China) was 50 nM, and that of si-WNK3 (RiboBio, China) was 100 nM. After a further 24 h, cells were harvested. The transfection efficiency of the siRNA and mimic was measured relative to the expression of a housekeeping gene by qRT–PCR.

lncRNAH19: siRNA: CCTCTAGCTTGGAAATGAA.

WNK3: siRNA: GACCGACAGTTGTTTCACA.

### Dual-luciferase reporter assay

HEK-293 T cells were seeded at 1.0 × 10^6^ cells/well in 35-mm Petri dishes 1 day before transfection. Cells were transfected with the miR-130-3p mimic and WNK3 wild-type plasmids/ mutant-type plasmids (2.5 μg) or H19 wild-type plasmids/ mutant-type plasmids (2.5 μg). The transfection reagents used were jetPRIME® and Polyplus-transfection (USA). Cells were incubated for 24 h and, when indicated, were analyzed with a Dual-Luciferase Reporter Gene Assay Kit (Beyotime, China) following the manufacturer’s instructions. The vector used for the dual-luciferase reporter was pmiR-RB-Report (RiboBio, China), which contains the Renilla luciferase (Rluc) reporter gene and firefly luciferase (Fluc) reporter gene. Rluc was used as the internal control.

### CCK-8 assay

Cell viability was evaluated using a Cell Counting Kit-8 (CCK-8, APExBIO, USA). Cells were seeded at 3 × 10^3^ cells/ well in 96-well plates. Cells were irradiated 24 h after transfection. After 24 h, 48 h, 72 h of treatment, 10 μL of CCK-8 reagent was added to every well for two hours. The optical density (OD) was measured at 450 nm.

### 5-Ethynyl-20-deoxyuridine (EdU) incorporation assay

The EdU incorporation assay was performed using a Cell-Light EdU DNA Cell Proliferation Kit (RiboBio, China). Images were acquired with a fluorescence microscope (Olympus, Japan).

### Colorogenic survival assay

After transfection and irradiation, the appropriate numbers of cells were seeded in triplicate in 60-mm Petri dishes. After 2 weeks of culture, cells were fixed with 4% polyformaldehyde and stained with 1% crystal violet. Colonies containing at least 50 cells were counted.

### Western blot analysis

Cells were lysed in RIPA buffer with protease and phosphatase inhibitors. The protein concentration was measured by a BCA assay kit (Thermo Scientific, USA). Lysates were denatured at 100 °C for 10 min and separated on a 10% or 15% SDS polyacrylamide gel. Proteins were transferred to PVDF membranes (Millipore, USA) and blocked with BSA (Solarbio, China) for 1.5 h. The antibodies used were specific for phosphorylated p38 (Immunoway, YP0338 1:1000), Bcl-2 (Gene Tex, GTX50413 1:500), Bax (Gene Tex, GTX56246 1:500), cleaved Caspase 3 (Gene Tex, GTX86900 1:1000), cleaved PARP (Affinity, AF7023 1:1000) and GAPDH (Gene Tex, GTX100118 1:5000). Western blot membranes are shown in Fig. 4D, E and F. All western blot signals were quantified using Image J software.

### Flow cytometry

Both A549 and H460 cells were seeded in 35-mm Petri dishes and were then irradiated with 6 Gy 24 h after transfection. After further incubation for 24 h, 48 h, and 72 h, cells were collected and stained with Annexin V and PI. In brief, cells were stained with an apoptosis kit (Roche, USA) following the manufacturer’s instructions. Data were analyzed with FlowJo v10.1.

### Statistical analysis

Statistical analyses were performed using GraphPad Prism software v7.0. Differences between paired samples were assessed using paired t-tests. Comparisons between treatment groups were made by Student’s t-test or ANOVA, as appropriate. All experiments were repeated three times. A significant difference was defined as p < 0.05.

## Results

### lncRNA H19 regulates the sensitivity of NSCLC cells to X-ray and carbon ion irradiation

**Fig. 1:**
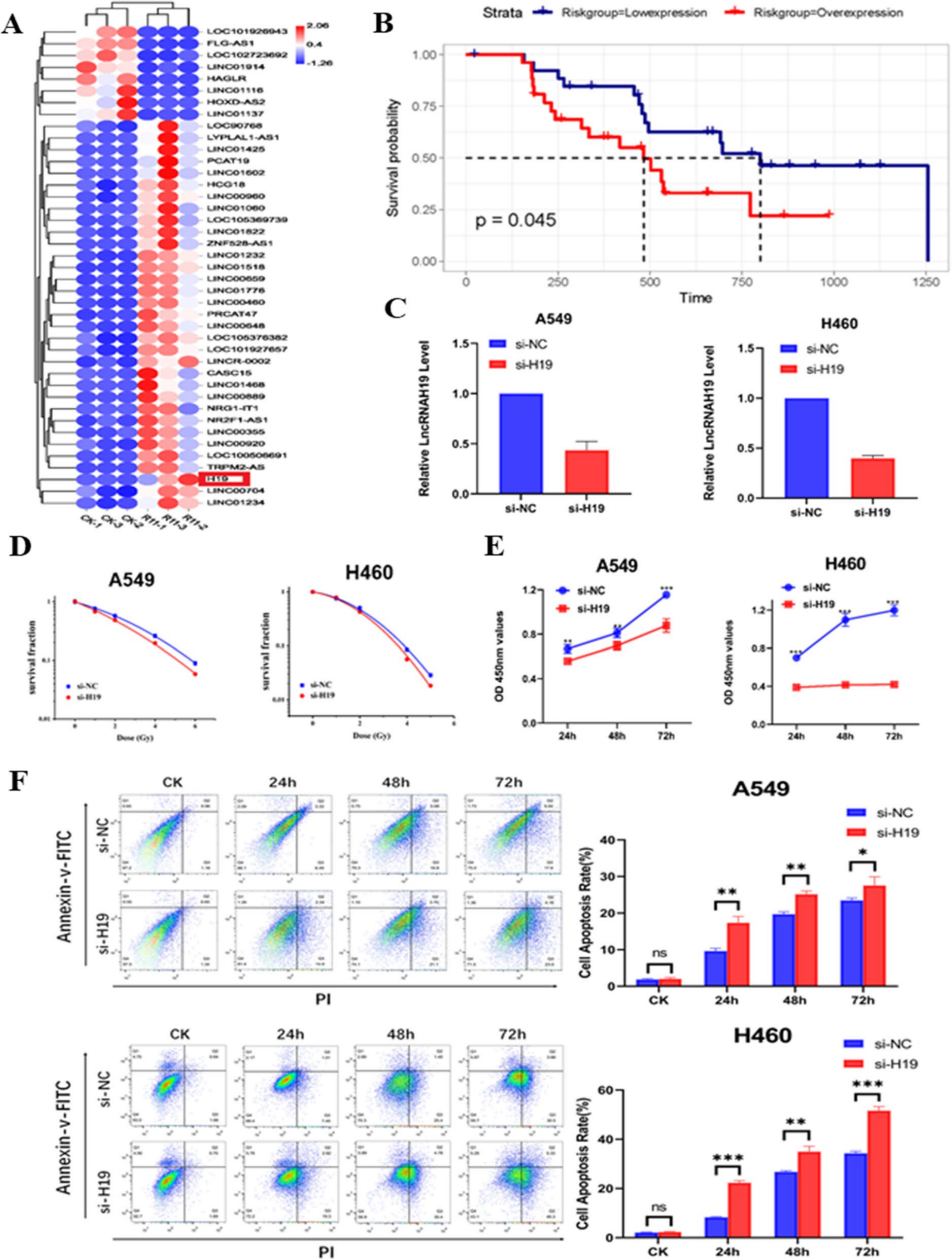
lncRNA H19 regulates the sensitivity of NSCLC cells to X-ray and carbon ion irradiation. **A** Differential lncRNA expression was found between A549 and radioresistant (A549-R11) cells. **B** The OS of NSCLC patients with different expression levels of lncRNA H19 after radiotherapy. **C** The transfection efficiency of H19 was measured by qRT–PCR. **D** Colony formation ability after H19 knockdown and irradiation. **E** Cell viability after H19 knockdown and irradiation was measured by a CCK-8 assay. **F** Flow cytometric analysis of apoptosis after H19 knockdown and irradiation. The data are presented as the mean ± SD values. *p < 0.05, **p < 0.01, ***p < 0.001 compared with the control group

In A549 cells and the compared radioresistant cells, the differentially expressed lncRNAs were investigated by high-throughput sequencing. The lncRNA H19 was upregulated in radioresistant NSCLC cells compared with A549 cells (Fig. 1A). The radioresistant cells were generated in our previous study [19]. According to the TCGA database (https://tcga-data.nci.nih.gov/tcga/), a high level of lncRNA H19 was related to poor survival in lung cancer patients who received radiotherapy (Fig. 1B). LncRNA H19 knockdown assays were conducted to elucidate the function of the lncRNA H19 in the radiosensitivity of NSCLC cells. First, the expression of the lncRNA H19 was downregulated after siRNA transfection in both A549 and H460 cancer cells. The inhibition efficiency was confirmed by qRT–PCR (Fig. 1C). Furthermore, lncRNA H19 knockdown combined with X-ray irradiation inhibited colony formation (Fig. 1D). Similarly, lncRNA H19 knockdown together with carbon ion irradiation inhibited the colony formation of A549 cells (Additional file 1: Fig. S1A). Compared with that of NC-treated cells, the proliferation of A549 and H460 cells was suppressed when they were treated with lncRNA H19 downregulation combined with 6 Gy irradiation (Fig. 1E). The EdU incorporation assay indicated that DNA synthesis was decreased after lncRNA H19 inhibition and irradiation (Additional file 1: Fig. S1B). When both A549 and H460 cells were treated with 6 Gy X-ray irradiation, the rates of apoptosis were significantly lower than those of the corresponding cells with lncRNA H19 knockdown (Fig. 1F). Taken together, these results revealed that lncRNA H19 inhibition sensitizes NSCLC cells to both X-ray irradiation and carbon ion irradiation.

### lncRNA H19 acts as a competing endogenous RNA by sponging miR-130a-3p

**Fig. 2:**
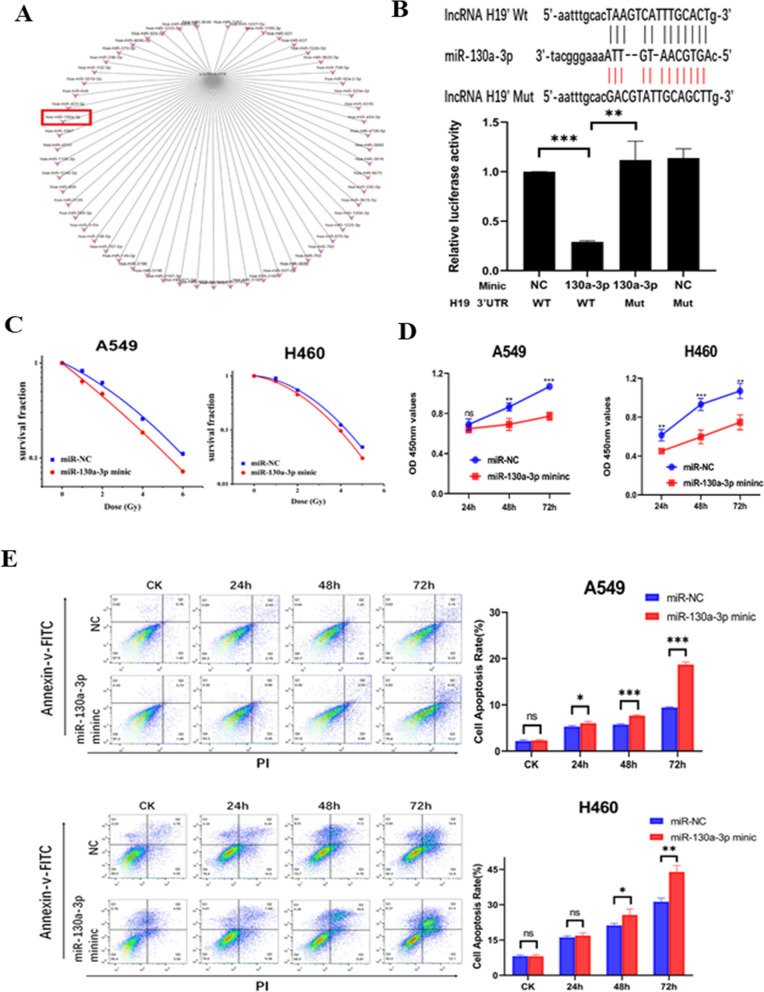
lncRNA H19 acts as a competing endogenous RNA by sponging miR-130a-3p. **A** Predicted miRNAs interacting with lncRNA H19. **B** Top: Binding site for miR-130a-3p in the H19 3′-UTR. Bottom: HEK-293 T cells were transfected with a miR-130a-3p mimic/NC and luciferase reporter vector containing the H19 3′-UTR (WT) or its mutant construct (Mut). The WT-NC value was set to “1.0”. **C** Colony formation ability after miR-130a-3p mimic transfection and irradiation. **D** Cell viability after miR-130a-3p mimic transfection and irradiation was measured by a CCK-8 assay. **E** Flow cytometric analysis of apoptosis after miR-130a-3p mimic transfection and irradiation. The data are presented as the mean ± SD values. *p < 0.05, **p < 0.01, ***p < 0.001 compared with the control group

According to online software (http://www.mirbase.org/), many miRNAs, including miR-130a-3p, can bind to the lncRNA H19 (Fig. 2A). As predicted by online lncRNA prediction software (http://starbase.sysu.edu.cn/), there was a potential interaction between H19 and miR-130a-3p. Dual-luciferase reporter assays were performed to confirm that H19 was the direct target gene of miR-130a-3p (Fig. 2B). Luciferase activity in the miR‐130a‐3p mimic + H19 WT group was lower than that in the NC groups and miR‐130a‐3p mimic + H19 Mut group. The two mutant plasmid groups had similar luciferase activities. After knockdown of lncRNA H19, the expression of miR-130a-3p was upregulated compared with that in NC-treated cells (Additional file 2: Fig. S2A). Collectively, the above data suggested that H19 could bind to miR-130a-3p. Moreover, after 24 h of transfection with the miR-130a-3p mimic, the expression of miR-130a-3p was significantly upregulated in both A549 and H460 cells (Additional file 2: Fig. S2B). Colony formation assays were used to explore whether miR-130-3p regulates the radiosensitivity of NSCLC cells. Overexpression of miR-130a-3p increased the radiosensitivity of NSCLC cells compared to negative control cells (Fig. 2C). The results of the CCK-8 assay revealed that the miR-130a-3p mimic significantly reduced cell viability compared with that in the control groups after irradiation (Fig. 2D). The EdU incorporation assay showed that DNA synthesis in NSCLC cells was decreased after miR-130a-3p mimic transfection and irradiation (Additional file 2: Fig. S2C). Furthermore, a flow cytometry assay was conducted to explore whether miR-130a-3p regulates the apoptosis of A549 and H460 cells. The miR-130a-3p mimic promoted the apoptosis of both A549 and H460 cancer cells after 6 Gy irradiation (Fig. 2E). These results indicated that lncRNA H19 acted as a competing endogenous RNA by sponging miR-130a-3p, which sensitized NSCLC cells to irradiation.

### WNK3 is the downstream target gene of miR-130a-3p

**Fig. 3:**
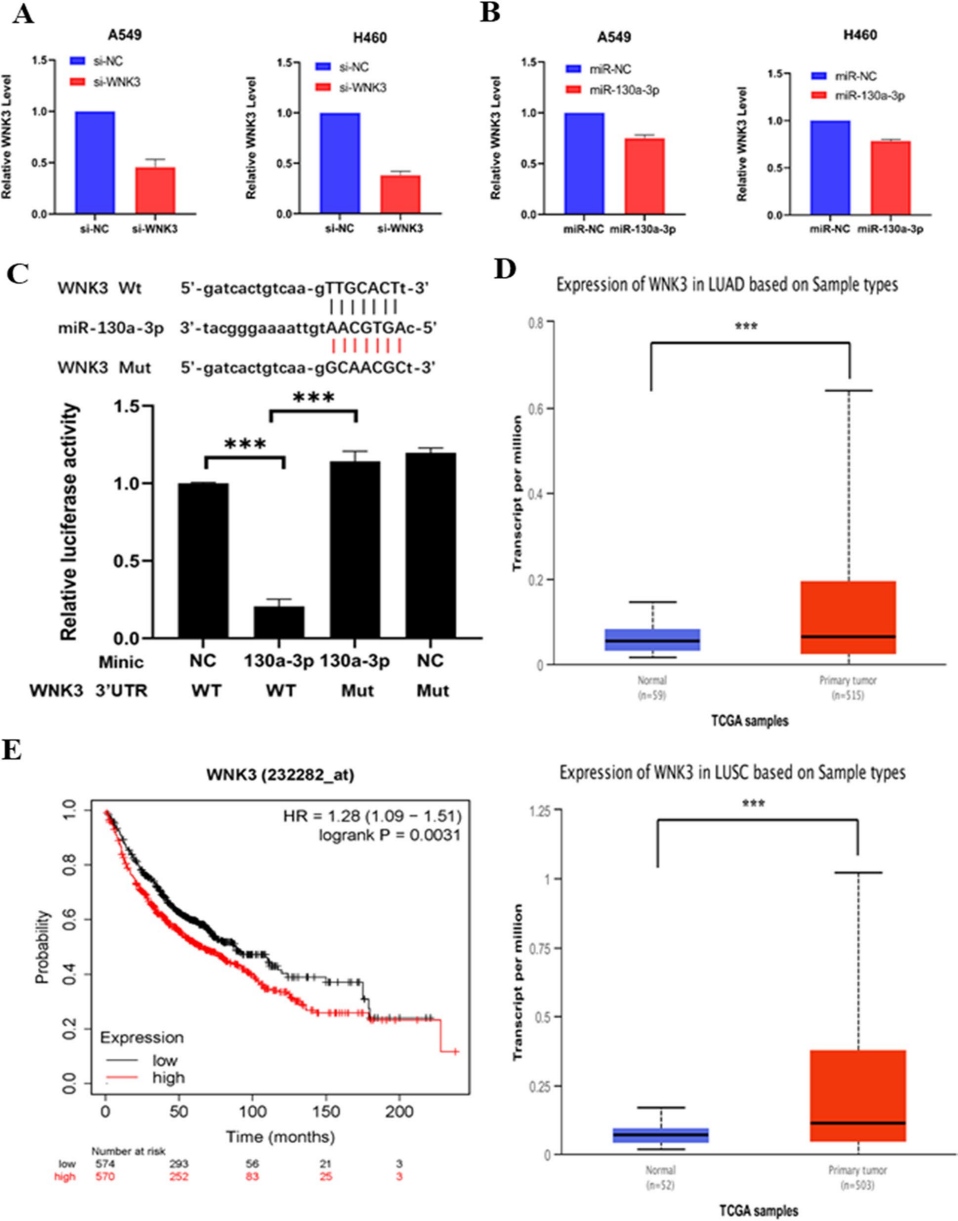
WNK3 is the downstream target gene of miR-130a-3p. **A** The transfection efficiency was measured by qRT–PCR. **B** Relative expression of WNK3 after transfection with the miR-130a-3p mimic. **C** Top: The binding site for miR-130a-3p in the WNK3 3′-UTR. Bottom: HEK-293 T cells were transfected with the miR-130a-3p mimic/NC and luciferase reporter vector containing the WNK3 3′-UTR (WT) or its mutant construct (Mut). The WT-NC value was set to “1.0”. **D** Top: Relative expression of WNK3 between normal tissue and tumor tissue in LUAD. Bottom: Relative expression of WNK3 between normal tissue and tumor tissue in lung squamous cell carcinoma (LUSC). **E** High levels of WNK3 in NSCLC samples were related to poor OS

An online database (http://www.targetscan.org/) was used to predict miR-130a-3p target genes. WNK3 was a candidate. First, the inhibition efficiency of WNK3 was determined by qRT–PCR in both A549 cells and H460 cells (Fig. 3A). The expression of suitable target genes should negatively correlate with the expression of the miRNA of interest. In NSCLC cells, when miR-130a-3p was overexpressed, WNK3 expression was downregulated compared with that in the NC cells (Fig. 3B). According to this result, WNK3 was a suitable target gene of miR-130a-3p. Hence, WNK3 was chosen for further validation. A dual-luciferase reporter assay was used to validate the direct binding between miR-130a-3p and WNK3 (Fig. 3C). Dual-luciferase reporter systems were constructed that included both wild-type and mutated miR-130a-3p recognition elements in the 3'- untranslated region (UTR) of WNK3. When the mixture containing the miR-130a-3p mimic and WNK3 wild-type reporter plasmid was cotransfected into HEK-293 T cells, luciferase activity was attenuated compared with that in the NC group and mutated plasmid groups. These findings confirmed that WNK3 was a target of miR-130a-3p. Another online database (http://ualcan.path.uab.edu) was used to analyze WNK3 expression. WNK3 was highly expressed in NSCLC samples (Fig. 3D). Additionally, online website analysis (http://kmplot.com) revealed that a high level of WNK3 in NSCLC samples was related to poor OS (Fig. 3E).

### WNK3 modulated the radiosensitivity of NSCLC cells and affected the P38 signaling pathway

**Fig. 4:**
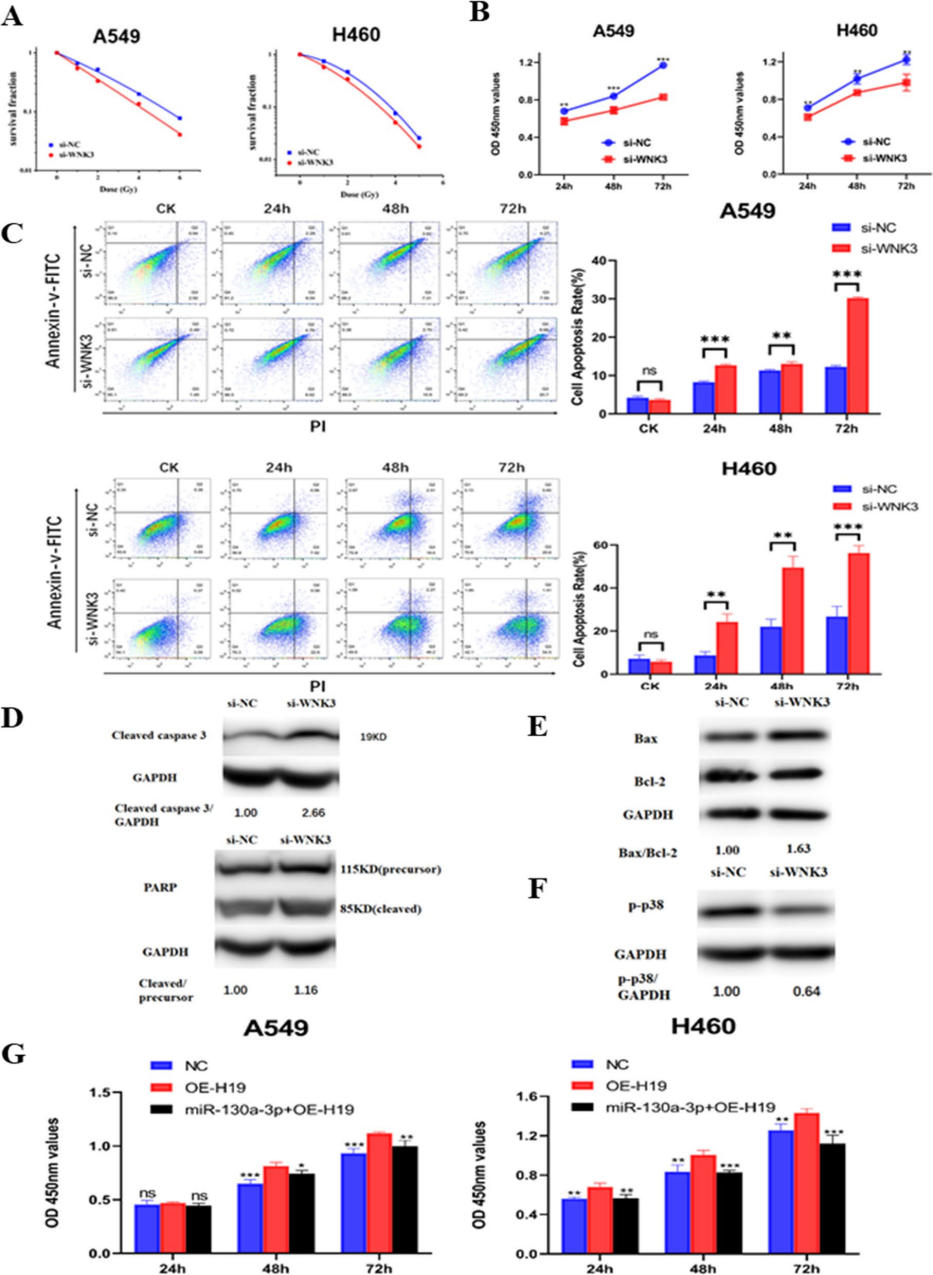
WNK3 modulated the radiosensitivity of NSCLC cells and affected the P38 signaling pathway. **A** Colony formation ability after WNK3 knockdown and irradiation. **B** Cell viability after WNK3 knockdown and irradiation was measured by a CCK-8 assay. **C** Flow cytometric analysis of apoptosis after WNK3 knockdown and irradiation. **D** The levels of (Top) cleaved caspase3 and (Bottom) PARP (85 kDa, cleaved; 115 kDa, precursor) in H460 cells after WNK3 knockdown and irradiation. The si-NC group was set to “1.00”. GAPDH was used as the control. **E** The expression of Bax and Bcl-2 after WNK3 knockdown and irradiation. The si-NC group was set to “1.00”. GAPDH was used as the control. **F** The expression of p-p38 in H460 cells after WNK3 knockdown and irradiation. The si-NC group was set to “1.00”. GAPDH was used as the control. **G** Cell viability after transfection with the NC or H19 overexpression plasmid or cotransfection with the H19 overexpression and miR-130a-3p mimic and treatment with irradiation was tested using a CCK-8 assay. The data are presented as the mean ± SD values. *p < 0.05, **p < 0.01, ***p < 0.001 compared with the control group

The function of WNK3 in the radiosensitivity of NSCLC cells was explored. Colony formation assays showed that WNK3 knockdown combined with X-ray irradiation inhibited colony formation (Fig. 4A). After 6 Gy X-ray irradiation, the WNK3 inhibitor dramatically suppressed the proliferation of NSCLC cells compared with NC-treated cells (Fig. 4B). The EdU incorporation assay indicated that the proliferation of both A549 and H460 cells was decreased after combined inhibition of WNK3 and irradiation (Additional file 3: Fig. S3). Additionally, WNK3 inhibition together with irradiation promoted the apoptosis of NSCLC cells (Fig. 4C). Taken together, these results revealed that WNK3, the downstream target gene of miR-130a-3p, modulated the radiosensitivity of both A549 and H460 cancer cells. Next, we analyzed the well-known proteins in the apoptotic signaling pathway by Western blotting. After cotreatment with WNK3 knockdown and X-ray irradiation, the level of cleaved caspase3 was dramatically increased compared with that in the NC group. The ratio of cleaved PARP/precursor PARP was just slightly elevated (Fig. 4D). Moreover, the ratio of Bax/Bcl-2 was increased in the WNK3 knockdown group (Fig. 4E). Previous studies have shown that WNK3 and p38 modulate the apoptotic response in HeLa cells [20, 21]. Hence, there has been speculation of whether this effect could occur in NSCLC cells. Consequently, the level of phosphorylated p38 (p-p38) was evaluated (Fig. 4F). WNK3 knockdown combined with 6 Gy irradiation decreased the level of p-p38. In addition, a rescue experiment was conducted to analyze whether the lncRNA H19 regulates radiosensitivity through miR-130a-3p in NSCLC cells. Both A549 and H460 cancer cells were transfected with the NC plasmid, lncRNA H19 overexpression plasmid or both the lncRNA H19 overexpression plasmid and the miR-130a-3p mimic, and were then exposed to 6 Gy X-ray irradiation. Overexpression of lncRNA H19 enhanced the radioresistance of NSCLC cells, and this effect was reversed by the miR-130a-3p mimic (Fig. 4G).

## Discussion

Radiotherapy is an important treatment modality with indications in all stages of NSCLC. However, it is underutilized. According to an evidence-based approach, it was estimated that seventy-seven percent of patients with lung cancer should receive radiotherapy [22, 23]. To achieve a better outcome of radiotherapy, radiation should be delivered to tumors at higher doses, which may lead to unacceptable side effects. Therefore, potential targets to help enhance radiosensitivity should be identified. NSCLC is characterized by alterations in multiple cellular pathways. Unsatisfactorily, most targeted treatments only act on a single pathway, which may lead to drug resistance. A single miRNA can affect the expression of multiple mRNAs, and each mRNA is, in turn, regulated by many miRNAs, forming a complex miRNA-mRNA network. Hence, it is hoped that drug resistance will be overcome by miRNA-targeted drugs in the near future. LncRNAs and miRNAs can regulate greatly varied processes through a competing endogenous RNA mechanism in numerous cancer types, which suggests that they may be used as therapeutic targets, biomarkers and prognostic indicators.

Radiosensitivity is modulated by lncRNAs in tumors. For example, lncRNA CRNDE/PRC2 targeting of p21 enhances radioresistance in NSCLC [24]. A recent study revealed that linc-SPRY3-2/3/4, a noncoding RNA on the Y chromosome, regulates radiosensitivity and affects apoptosis and cell viability in NSCLC [25]. The lncRNA PVT1, defined as a factor indicating poor prognosis, enhances radioresistance by regulating apoptosis in nasopharyngeal carcinoma [26]. The interaction between the lncRNA H19 and miR-193a-3p regulates the radiosensitivity of hepatocellular carcinoma cells [27]. Hence, the identification of radiosensitivity-related molecular mechanisms may help to improve the efficacy of radiotherapy and increase its antitumor effect. In this study, the lncRNA H19 was shown to be highly expressed in radioresistant NSCLC cells. LncRNA H19 inhibition sensitized NSCLC cells to both X-ray irradiation and CIRT. This result seemed, at least in part, to confirm our hypothesis that the lncRNA H19 can modulate radiosensitivity in NSCLC. In recent years, several advances have been made in H19-related drug development. BC-819, a DNA plasmid, is a potential therapeutic approach for cancers that overexpress the H19 gene. BC-819 was safe and well tolerated for the treatment of unresectable pancreatic cancer, recurrent ovarian cancer and bladder cancer. Inspiringly, BC-819 combined with chemotherapy may enhance the antitumor therapeutic efficacy [28–30]. Therefore, inspiring results with BC-819 combined with radiotherapy can be expected in the near future.

MiRNAs indirectly contribute to protein coding. However, once miRNAs bind to the 3’ UTR of an mRNA, the mRNA is degraded or cannot be translated [31]. MiRNAs have been demonstrated to influence treatment outcomes and to predict prognosis in cancer. The lncRNA H19, as an endogenous sponge, can directly target miR-130a-3p. The dual-luciferase reporter assay results seemed to confirm this idea. Moreover, miR-130a-3p inhibited cell proliferation, induce apoptosis and determined radiosensitivity in NSCLC. Similarly, miR130a serves as a tumor suppressor in NSCLC and cutaneous squamous cell carcinoma. Low expression of miR130a was found to be related to the poor 5-year OS of NSCLC patients. Mechanistically, miR-130a downregulates the expression of KLF3 to inhibit the growth of NSCLC cells [32]. The miR-130a level was found to be low in primary NK cells of NSCLC patients. The killing ability of NK cells was enhanced by the biological function of miR-130a [33]. Additionally, through in vivo and ex-vivo experiments, miR130a was found to play a tumor-suppressive role in cutaneous squamous cell carcinoma [34].

Radiotherapy is applied in the treatment of various types of tumors, and many miRNAs associated with the response to radiation treatment can be used to predict the efficacy of radiotherapy. For example, the radiosensitivity of cervical cancer in vitro was found to be promoted by miR-22, via promotion of apoptosis [35]. MiR-27 was found to increase the sensitivity of NSCLC cells to radiotherapy via homologous recombination-mediated DNA repair and was recognized as a therapeutic target in NSCLC [10]. In recent years, several preclinical trials targeting miRNAs have been initiated. Miravirsen, an antagomiR of miRNA-122 and the first miRNA-targeted drug, was investigated and found to compromise HCV replication [36]. TargomiR, a miR-15/107 miRNA mimic, was shown to be well tolerated and safe in patients with recurrent thoracic cancer in a phase I study. Interim data indicated that six patients received the eight-week treatment protocol, and five of them achieved disease control [37]. Similarly, miR-155 regulates multiple pathways, including the JAK/STAT, MAPK/ERK and PI3K/AKT pathways. Cobomarsen, an antagomiR of miR-155, underwent a phase I clinical trial for mycosis fungoides, the most common type of cutaneous T‐cell lymphoma [38]. Taken together, these observations indicate that targeted miRNA drugs in combination with radiotherapy may be a promising antitumor approach.

More specifically, WNK3, an important factor in many pathways, functions as an accelerator of cancer. A dual-luciferase reporter assay was used to confirm that miR-130a-3p contains specific binding sites for WNK3 (Fig. 3C). The mechanism of action of WNK3 involves procaspase-3 and heat shock protein 70. Due to suppression of WNK3, the apoptotic response is promoted and the activation of caspase-3 is accelerated in HeLa cells [20]. In this study, WNK3 inhibition combined with irradiation markedly increased the cleaved caspase3 level compared with that in NC-treated cells. The antitumor effect of induced tumor cell apoptosis is the main mechanism of radiotherapy. Bax, bcl-2 and PARP are well-known markers of the apoptotic signaling pathway. Our study revealed that WNK3 inhibition combined with X-ray irradiation increased the ratio of Bax/Bcl-2 and cleaved PARP /precursor PARP. In addition, WNK3 functions as a “bad boy” because it promotes invasion in glioma [39]. However, the function of WNK3 in NSCLC is completely unknown. In our study, WNK3 was highly expressed and was correlated with poor prognosis in NSCLC. Functionally, WNK3 inhibition promoted apoptosis and increased the radiosensitivity of NSCLC cells to X-ray irradiation. Silencing of the lncRNA H19 and WNK3 promoted the apoptotic response and enhanced radiosensitivity in NSCLC cells. The same effects were found for miR-130a-3p mimic. Moreover, lncRNA H19 served as a sponge of miR-130a-3p, as confirmed by the dual-luciferase reporter assay. Additionally, the binding relationship between miR-130a-3p and WNK3 was determined. Hence, the lncRNA H19–miR-130a-3p–WNK3 axis exists and regulates the radiosensitivity of NSCLC cells.

In addition, p38 pathways are activated by various environmental and genotoxic stress agents and regulate various cellular functions, including apoptosis, proliferation, migration and so on [40, 41]. Similar biological functions were found in the present study. After 6 Gy irradiation and WNK3 knockdown, the level of phosphorylated p38 was decreased. With the expanding development of targeted agents, ralimetinib (LY2228820), an inhibitor of p38, has been developed and applied clinically. A randomized controlled trial with 118 patients revealed that ralimetinib in combination with gemcitabine and carboplatin could improve the progression-free survival (PFS) of patients with recurrent ovarian cancer [42]. Moreover, encouraging results were obtained, including for ralimetinib combined with radiotherapy plus temozolomide, in the treatment of glioblastoma [43], and for ralimetinib combined with tamoxifen to treat advanced breast cancer [44]. Combined with these results, our study indicates that radiotherapy combined with ralimetinib could be considered a modality for NSCLC in future clinical trials.

Conceivably, the identification of more mechanisms of lncRNAs that promote radiosensitivity or radioresistance, especially related to their function as biomarkers, prognostic indicators and therapeutic targets, are needed in different cancer types. LncRNAs can bind to miRNAs and serve as ceRNAs to modulate radiosensitivity, and we focused on lncRNA H19–miR-130a-3p–WNK3 axis in NSCLC in this study. Powered by advanced RNA-based therapeutics, in the distant future, radiosensitivity-related RNA-targeting agents can be developed to enhance the anticancer effect of radiotherapy in NSCLC.

## Supplementary Information

**Additional file 1.** (**A**): Colony formation ability after H19 knockdown and carbon-ions irradiation in A549 cells. (**B**): The EdU incorporation assay after lncRNA H19 knockdown and irradiation.

**Additional file 2.** (**A**): Relative expression of miR-130a-3p after transfection with the si-H19. (**B**): The transfection efficiency was measured by qRT–PCR. (**C**): The EdU incorporation assay after miR-130a-3p mimic and irradiation.

**Additional file 3.** The EdU incorporation assay after WNK3 knockdown and irradiation.

## Data Availability

The datasets generated and analyzed during the current study are publicly available from the following online databases: Mirbase database (http://www.mirbase.org/); (http://www.targetscan.org/); TCGA (https://tcga-data.nci.nih.gov/tcga/); starBase v2.0 (http://starbase.sysu.edu.cn/); UALCAN (http://ualcan.path.uab.edu); Kaplan–Meier Plotter (http://kmplot.com).

## Abbreviations

NSCLC: Non-small cell lung cancer
CCK8: Cell counting kit-8
Edu: 5-Ethynyl-20-deoxyuridine
WNK3: With-no-lysine kinase 3
CIRT: Carbon ion radiotherapy
LET: Linear energy transfer
LC: Local control
OS: Overall survival
SBRT: Stereotactic body radiation therapy
FBS: Fetal bovine serum
miRNAs: MicroRNAs
lncRNAs: Long noncoding RNAs
LUAD: Lung adenocarcinoma
LUSC: Lung squamous cell carcinoma
SNPs: Single-nucleotide polymorphisms
FBS: Fetal bovine serum
OD: Optical density
UTR: Untranslated region
mRNA: Messenger RNA
PFS: Progression-free survival
