# Supplementary figures and images for: Silencing of the lncRNA H19 enhances sensitivity to X-ray and carbon-ions through the miR-130a-3p /WNK3 signaling axis in NSCLC cells
Source: Cancer Cell Int. 2021 Dec 4;21:644. doi: 10.1186/s12935-021-02268-1 (PMC8642868; doi:10.1186/s12935-021-02268-1)

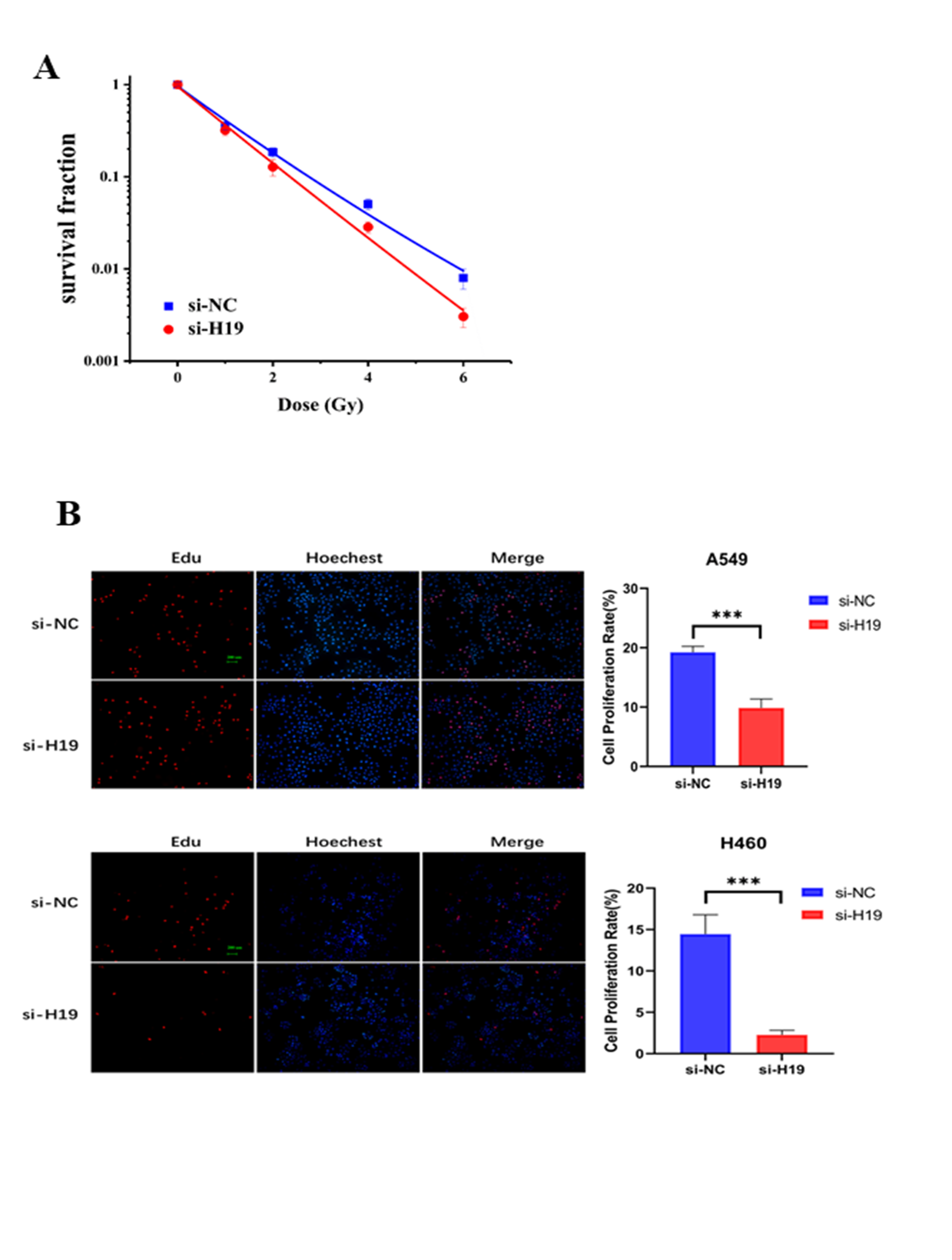

Supplement: Supplementary file 1 — Additional file 1. (A): Colony formation ability after H19 knockdown and carbon-ions irradiation in A549 cells. (B): The EdU incorporation assay after lncRNA H19 knockdown and irradiation. [file 12935_2021_2268_MOESM1_ESM.tif]

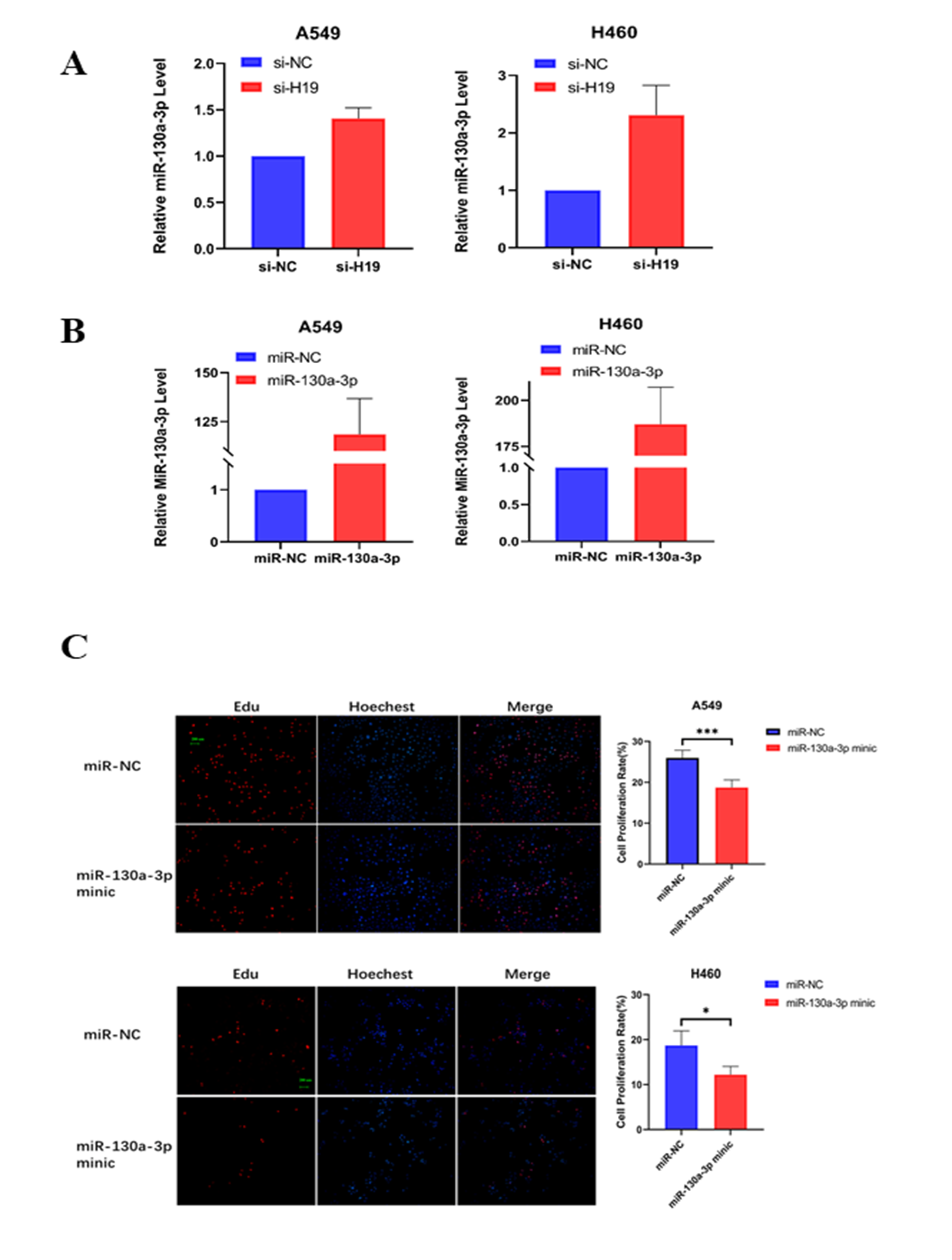

Supplement: Supplementary file 2 — Additional file 2. (A): Relative expression of miR-130a-3p after transfection with the si-H19. (B): The transfection efficiency was measured by qRT–PCR. (C): The EdU incorporation assay after miR-130a-3p mimic and irradiation. [file 12935_2021_2268_MOESM2_ESM.tif]

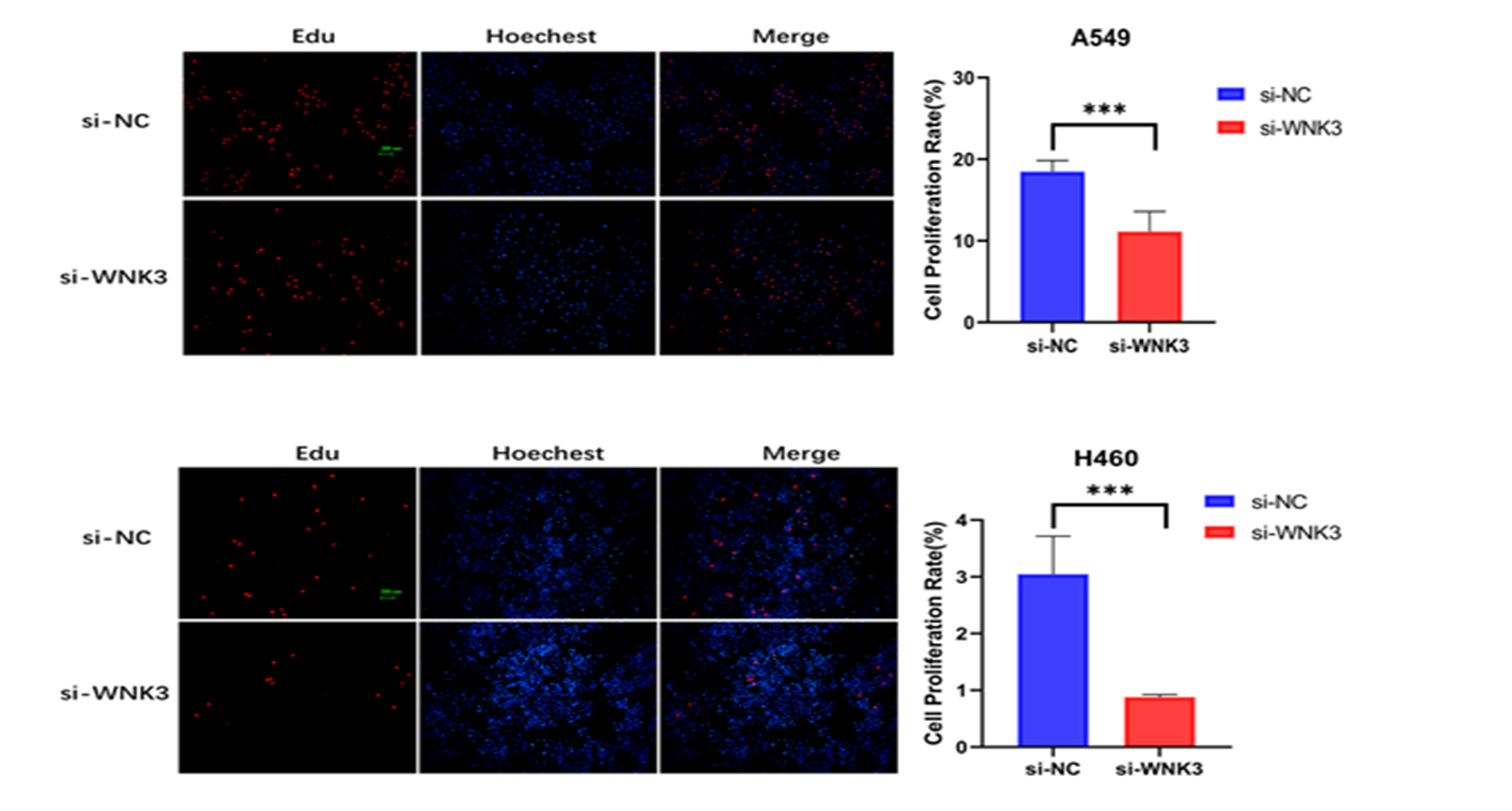

Supplement: Supplementary file 3 — Additional file 3. The EdU incorporation assay after WNK3 knockdown and irradiation. [file 12935_2021_2268_MOESM3_ESM.tif]
